# The Framework for Responsible Research With Australian Native Plant Foods: A Food Chemist's Perspective

**DOI:** 10.3389/fnut.2021.738627

**Published:** 2022-01-14

**Authors:** Selina Fyfe, Heather E. Smyth, Horst Joachim Schirra, Michael Rychlik, Yasmina Sultanbawa

**Affiliations:** ^1^Queensland Alliance for Agriculture and Food Innovation (QAAFI), Health and Food Sciences Precinct, The University of Queensland, Brisbane, QLD, Australia; ^2^Centre for Advanced Imaging, The University of Queensland, Brisbane, QLD, Australia; ^3^School of Environment and Science, Griffith University, Nathan, QLD, Australia; ^4^Griffith Institute for Drug Discovery, Griffith University, Nathan, QLD, Australia; ^5^Chair of Analytical Food Chemistry, Technical University of Munich, Freising, Germany

**Keywords:** native Australian plant foods, responsible research, policy, ethical guidelines, framework, Australia

## Abstract

Australia is a rich source of biodiverse native plants that are mostly unstudied by western food science despite many of them being ethnofoods of Australian Indigenous people. Finding and understanding the relevant policy and legal requirements to scientifically assess these plants in a responsible way is a major challenge for food scientists. This work aims to give an overview of what the legal and policy framework is in relation to food chemistry on Australian native plant foods, to clarify the relationships between the guidelines, laws, policies and ethics and to discuss some of the challenges they present in food chemistry. This work provides the framework of Indigenous rights, international treaties, federal and state laws and ethical guidelines including key legislation and guidelines. It discusses the specific areas that are applicable to food chemistry: the collection of plant foods, the analysis of the samples and working with Indigenous communities. This brief perspective presents a framework that can be utilized by food chemists when developing responsible research involving plant foods native to northern Australia and can help them understand some of the complexity of working in this research area.

## Introduction

Australia is a rich source of biodiverse native plants that are mostly unstudied scientifically despite many of them being ethnofoods eaten by Australia's Indigenous people. Australia is one of the world's 17 megadiverse countries of flora and fauna with 93% of flowering plants occurring only in it (1). Over the past 10 years native Australian plant foods have become more desired and accepted in mainstream Australian culture and the past five years has seen many becoming common as valued ingredients in food products on supermarket shelves, in gourmet foods and on restaurant menus where they are usually an ingredient featured in the name and marketing of the product. Researching native Australian plant foods offers the possibility of more of them being added to the Australian food supply chain and enabling Indigenous communities to be an important part of this.

Relatively few Australian native plant foods have been studied by western food science to understand their nutritional or functional components or to understand how they can be added to the native food value chain and made commercially available. Some of those that have been studied have been shown to have high values in key nutrients (2, 3) and excellent food preservation properties (4) as well as unique and interesting flavors (5). The Kakadu plum is now used in the Queensland prawn industry as a natural preservative to increase the shelf life of cooked chilled prawns (4).

Food chemists study foods to understand their composition and properties and to identify ways they can be used as food products and as ingredients in other food products. For food chemists to do responsible research on these plant foods they must comply with international, national and state policies, treaties and laws as well as uphold Indigenous rights and obtain the samples and conduct the research in a legal and ethical manner. Finding and understanding all of these requirements is a major challenge.

The European Union's Rome Declaration on Responsible Research and Innovation in Europe (6) defines responsible research and innovation as “the on-going process of aligning research and innovation to the values, needs and expectations of society.” It involves engagement of all societal actors, ethics, respecting fundamental rights, governance through policy (7), having research integrity and it should make science more sustainable, ethical and socially beneficial (8). The aim of this paper is to collate the current relevant key legal and policy requirements into a framework for food chemists to study native Australian plants in a responsible way, and to comment on some of the challenges. This brief perspective focuses only on the perspective of food chemists and does not include chemistry related to medicine or pharmacology. It does not include responsible innovation which would require legal analysis of intellectual property laws and legal contracts. It is not a framework for product development or commercialization which would have other requirements including, but not limited to, different types of permits with different conditions and permission requirements, benefit sharing agreements, agreements around branding and market access, the traditional or novel status of the plant food with Food Standards Australia New Zealand and various other regulatory requirements. It is not a legal analysis by lawyers but a perspective of food chemists.

## Food Chemistry and Australian Native Plant Foods

Food chemistry aims to analyze the composition and properties of food and the compositional changes that occur during handling, processing and storage of food (9). Food chemistry studies of Australian native plant foods include nutrition profiles, flavor chemistry, compounds that give antioxidant and antimicrobial properties, chemical and metabolite composition, compounds that impact safety of these as foods and changes that occur during storage or processing. Recent studies include the nutritional profiling of wattle seeds (10, 11), green plum (3), Kakadu plum kernels (12) and gumby gumby leaves (13); the chemical and metabolite composition of Kakadu plum (2, 14), finger lime, native pepperberry and Davidson's plum (15) and Tasmanian pepper (16); the antioxidant properties of infusions of gulban, anise myrtle and lemon myrtle leaves (17), Kakadu plum extracts (18), wattle seeds (11), the tuckeroo (19), Illawarra plum, Kakadu plum, muntries and native currant (20); and analysis of storage of lemon myrtle, anise myrtle and Tasmanian pepper leaf (21) as well as drying methods of Kakadu plum (22). Food chemistry has the potential to be used in the provenance, traceability and authenticity of native Australian plant foods. Food chemistry studies including metabolomics have been used to assess the authenticity of the geographical locations of other food products (23–25). Metabolomics can be used to give both a fingerprint of the “terroir” metabolome and to identify key compounds to use as biomarkers for specific locations (25, 26) which can be used to guarantee the origins of a product and to protect against food fraud (23). One study of Kakadu plums using attenuated total reflectance mid infrared spectroscopy has shown that adulteration with synthetic ascorbic acid can be detected (27). Further food chemistry analysis in the near future is likely to include more of these types of profiling of other native plant foods and more in-depth chemical and metabolite composition analysis of previously studied ones. This analysis is important for adding these foods as ingredients into other foods, for increasing their use throughout Australia, and for enabling sustainable Indigenous agri-businesses to be created. To conduct these types of food analysis, food chemists need to ensure they understand how to do them responsibly, by obtaining food samples in a legal and responsible way, only doing analysis they are permitted to do, and with responsible and ethical involvement of Indigenous communities.

## The Framework for Responsible Research

**Figure 1:**
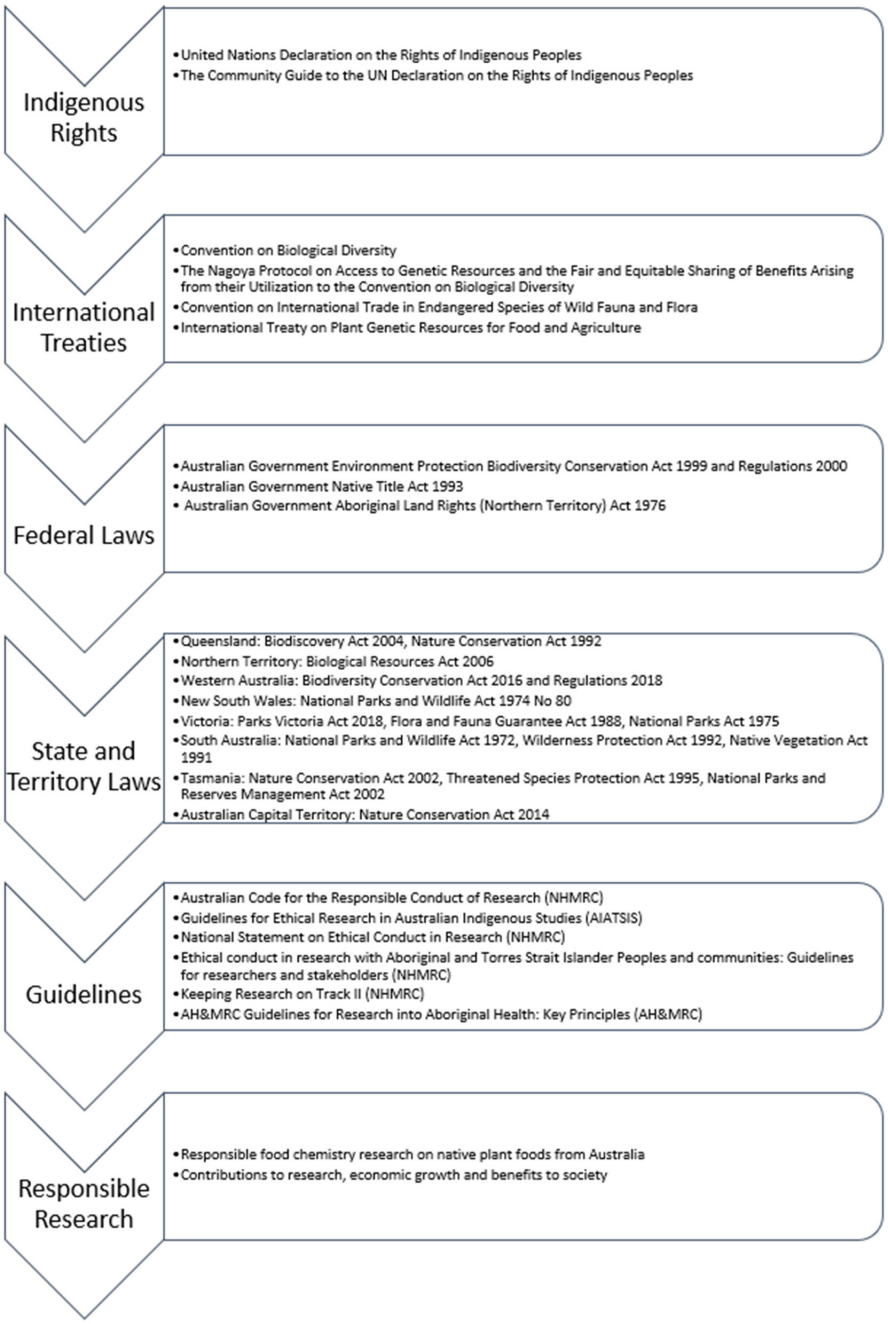
The framework for responsible research for food chemists working with Australian native plant foods. This framework contains key legislation; however, other laws and guidelines may also be relevant.

The framework for responsible research presented in Figure 1 was compiled by finding and reading each individual element of it then connecting and collating these together into a framework. The *Australian Code for the Responsible Conduct of Research* (28) states the principles of responsible research are honesty, rigor, transparency, fairness, respect, recognition, accountability and promotion. Burget et al. (29) describe the conceptual dimensions of responsible research as inclusion, anticipation, responsiveness, reflexivity, sustainability and care. The rights, treaties, laws and policies in the framework provide the governance measures and ethical guidance to enable food chemists to do responsible research on native Australian plant foods.

## Rights to Uphold: Indigenous Rights

The rights of Indigenous people add a dimension of inclusion to responsible research. Engagement of all stakeholders is considered essential for innovation to be sustainable, acceptable and desirable (6), and upholding the rights of Indigenous people means engaging with them and recognizing their right to be engaged in research (28). The *United Nations Declaration on the Rights of Indigenous Peoples* (30) and the Australian *The Community Guide to the UN Declaration on the Rights of Indigenous Peoples* (31) have determined how these rights relate to Indigenous Australians in this context. Article 31 states Indigenous Australians have “the right to maintain, control, protect, and develop their cultural heritage, traditional knowledge and traditional cultural expressions, as well as the manifestations of their sciences, technologies and cultures, including human and genetic resources, seeds, medicines, knowledge of the properties of fauna and flora…” etc. (30). Aboriginal and Torres Strait Islander communities in Australia have knowledge about these plants such as which ones are foods, when they are available, what should not be eaten, and how they should be processed to be made safe or edible (32–34). This extensive plant knowledge has been declared one of the important economic resources of Aboriginal Australians (35). The UN Declaration indicates research on Australian native plant foods used by Indigenous people should involve Indigenous people in the research. This complies with Articles 21 and 32 by giving Indigenous Australians an opportunity to improve their economic conditions and the right to determine and develop strategies for the development or use of their lands and other resources. In addition, such a research partnership allows compliance with Article 29, as Indigenous Australians have the right to the productive capacity of their land. It provides opportunities for communities to produce food from their land, support and create new businesses and develop the economic status of the community which will contribute to societal goals as well as economic growth (36, 37).

## Treaties to Comply With: International Treaties

International treaties provide requirements at an international level and protection against the threat of genetic material of native plant species being taken and domesticated in other countries (38). The international *Convention on Biological Diversity* (CBD) was put in place to help with conservation and sustainable use of biological diversity and to ensure there is benefit sharing of genetic resources (39). Article 15 relates to accessing genetic resources and it changed the authority to access natural resources from the “common heritage of mankind” to being that of the national government who now have sovereign rights over natural resources (39, 40) and states access must be on “mutually agreed terms,” that “prior informed consent” should be obtained and there should be “sharing in a fair and equitable way the results of research and development and the benefits arising from the commercial and other utilization of genetic resources with the Contracting Party providing such resources.” It is from this part of the CBD that the federal, state and territory laws for permits and benefit-sharing agreements for collection and study of native plants has been derived.

*The Nagoya Protocol* (41) is supplementary to the CBD and is not ratified in Australia (42), but must be complied with if Australian food products or samples are exported to countries that have ratified it. A more detailed explanation of this in relation to Australian native plant foods is given by Adhikari et al. (43). The *Bonn Guidelines* were written to operationalize the relevant provisions in the CBD (44). It gives guidelines about gaining prior informed consent to access genetic resources and basic guidelines of what should be considered in mutually agreed terms.

The *Convention on International Trade in Endangered Species of Wild Fauna and Flora* (CITES) (45) contains a list of endangered wild fauna and flora that CITES regulations relate to. The *International Treaty on Plant Genetic Resources for Food and Agriculture* recognizes that plant genetic resources are depended on for food and agriculture and that most plants that are farmed are being done in places other than where they originated (46). It addresses benefit sharing of plants used for food and agriculture (Article 1), and its purpose is partly to promote the conservation and food production of wild plants by supporting the efforts of local and Indigenous communities (Article 5) and to recognize the contribution they make in conservation and development of plant genetic resources (Article 9).

## Laws to Obey: Federal, State and Territory Laws

Article 15 of the CBD recognizes that states have the sovereign right over their natural resources and national governments have the authority to grant access to them. Governance of biological resources are within the respective jurisdiction of each state, territory, or the Commonwealth government in Australia who all have separate and differing laws and requirements. Figure 1 contains a list of the key federal and state Acts related to research on native Australian plants (47–63), although other Acts will also be relevant. These Acts enforce the international treaties and Indigenous rights and bring in the appropriate and legal ways to uphold these. They state what land they apply to and how they apply across ownership of land. They use permits and authorizations as a means of ensuring appropriate approval has been given for plant or fruit collection and that the “prior informed consent” required by the CBD has been given from both the government and from owners or title holders of the land. In some circumstances benefit sharing agreements are required to ensure the CBD requirement for “sharing in a fair and equitable way the results of research and development” does occur (39). Some Acts have other relevant requirements for the traceability of samples including for them to be botanically identified by a Herbarium, sampling and storage requirements, the requirement to keep records of the samples and the need for reporting on disposal of plant material.

The federal government has other laws relevant to collecting samples in conjunction with Indigenous communities including the *Native Title Act 1993* (64) and the *Aboriginal Land Rights (Northern Territory) Act 1976* (65) and some states have other relevant Acts too, not included in Figure 1. The states add information and requirements in relation to the collection of plant samples on land under Indigenous rights or native title, for example, in NSW the *National Parks and Wildlife Act 1974 No 80* (49) recognizes the cultural significance of certain lands to Aboriginal people (s71D) and details the requirements around Aboriginal land, objects and places.

## Guidelines to Follow: Ethics Guidelines

Responsible research involves working in an ethical way with all stakeholders on a project. Ethics approval is mandatory when doing research with human subjects, but the need to obtain approval from an ethics committee for research involving native Australian plant foods may not be as apparent to this research area. The ethics guidelines contribute ways of acting responsibly and upholding Indigenous rights when working and engaging with Indigenous communities. They give contextually appropriate ways of acting and thinking in an ethical manner, to help researchers understand their desire, motivation and the cultural narrative, and help them to act with moral judgement as a way to impede good intentions from having bad effects (66).

**Figure 2:**
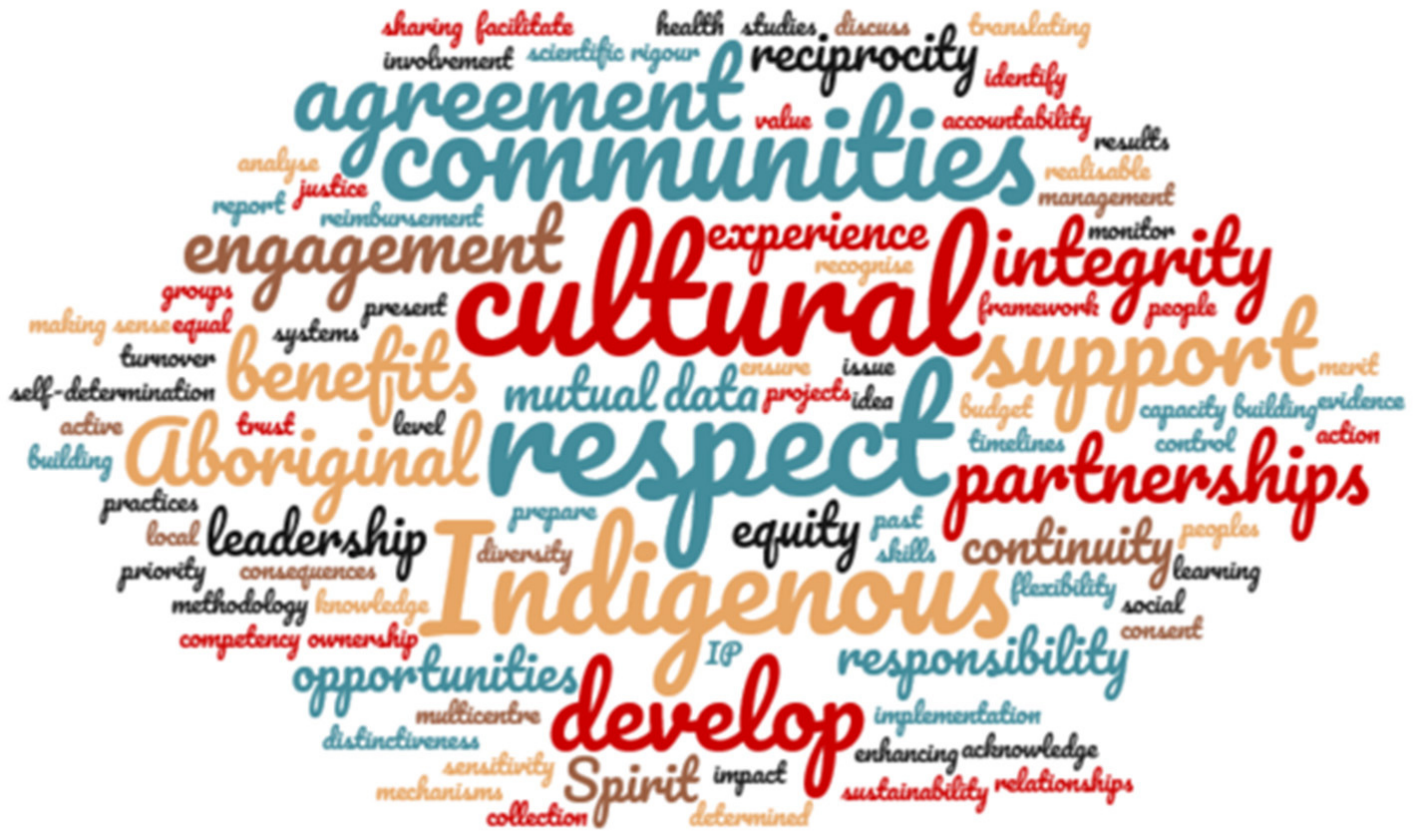
A word cloud summary of the principles and core values of the ethical guidelines relevant to the study of native Australian plants. The guidelines used were *AIATSIS Code of Ethics for Aboriginal and Torres Strait Islander Research* (67), the *National Statement on Ethical Conduct in Human Research* section 4.7 (28), *Ethical conduct in research with Aboriginal and Torres Strait Islander Peoples and communities: Guidelines for researchers and stakeholders* (70), *Keeping Research on Track II* (69), the *AH&MRC Guidelines for Research into Aboriginal Health: Key Principles* (71) and *Ten principles relevant to health research among Indigenous Australian populations* (72). The more frequently a key word is used in the respective text, the larger in size it is presented. This word cloud was made using WordClouds.com (Zygomatic).

When working with Indigenous community members researchers are required to comply with the *AIATSIS Code of Ethics for Aboriginal and Torres Strait Islander Research* (67). One of the functions of the Australian Institute of Aboriginal and Torres Strait Islander Studies (AIATSIS) is to provide ethical guidance for research under the *Australian Institute of Aboriginal and Torres Strait Islander Studies Act 1989* (68). The guidelines for ethical research have the principles of Indigenous self-determination, Indigenous leadership, impact and value and of sustainability and accountability. The National Health and Medical Research Council (NHMRC) *Keeping Research on Track II* (69) contains useful and practical ways and guidance for researchers in having Indigenous communities involved in research. Other relevant ethical guidelines are given in Figure 1 and also provide useful information about ethical research in this area. The word cloud in Figure 2 acts as a summary of the ethical guidelines and shows the main themes and terms which appear most frequently in the guidelines and was made using a method slightly modified from Maramba et al. (73).

The two most obvious themes in the ethics guidelines, as shown in Figure 2, are respect and cultural. Respect in the NHMRC guidelines relates to respect for Indigenous people and their knowledge (28, 70). Jamieson et al. (72) refers to respect as a mutually respectful partnership framework which comes from an open and transparent relationship. Culture is mentioned in a number of contexts including cultural continuity (69, 70), cultural distinctiveness (28), cultural competency (70), cultural expression (74) and cultural sensitivity (71).

There are general guidelines for ethical and responsible conduct of research which must also be complied with when conducting native plant food research including the *Australian Code for the Responsible Conduct of Research* (28), the *Statement on Consumer and Community Involvement in Health and Medical Research* (75) and, in Queensland, the *Queensland Biotechnology Code of Ethics* (76).

## Discussion

The framework above shows the complexity involved in scientifically studying Australian native plant foods. One challenge within the framework is for food chemists to understand if their planned work will be classed as biodiscovery. The definitions of “biodiscovery” and the related “bioprospecting” are different between the Acts, and definitions are not written with scientific detail. Overall, there is a lack of clarity within these laws about what it means to study chemical composition which could leave them open to multiple interpretations in relation to food chemistry analysis and potential commercial use. Providing guidelines and assistance to scientists in this field of research is essential if a consistent approach is desired. The definitions should clearly state if they apply only to novel or previously undiscovered compounds, or if they apply only to new commercial uses. Current definitions that state they apply to research relating to “biochemical compounds” do not acknowledge that all plant material is made up of chemical compounds including many important but common nutrition and safety compounds. Future policy development to make these definitions clear and interpretable by scientists from various relevant disciplines is highly recommended to facilitate equitable research compliance in this area.

Australian state and federal governments each endorsed a “Nationally consistent approach for access to and the utilization of Australia's native genetic and biochemical resources” on the 11th Oct, 2002 (77), agreeing there should be one approach across all of Australia. However, there are inconsistencies in the land they relate to, the definitions of biodiscovery, the access requirements, and the timing and need for benefit sharing agreements. A nationally consistent approach would help reduce confusion and prevent intentional and unintentional biopiracy from occurring (43).

The need for a benefit-sharing agreement is based on Article 15 of the CBD (39). As part of the Nationally Consistent Approach signed by Australian states it was intended that model contracts and dictionaries of contractual terms be developed for the benefit-sharing agreements so biodiscovery could be facilitated with a certainty of the process (77). The Australian Government has stated that the CBD objective of fair and equitable benefit sharing has been the most difficult to implement (40). Other challenges for food scientists in relation to benefit sharing agreements are understanding and agreeing on what fair and equitable may mean for different parties; working with multiple access providers; what to do when native plants grow over multiple locations; valuing non-monetary and intangible benefits; knowing how to provide reasonable support when handing a remote Indigenous community a legal document; and knowing what reasonable benefit sharing is in regards to Indigenous people's knowledge.

Food chemists collecting samples need to obtain the permits and authorizations required for the specific place where they intend collecting samples and need to understand the requirements for each place they collect from as this varies throughout Australia. This brief perspective shows some of the complexity involved in planning to study native Australian plant foods, before they can even be collected or scientifically studied. Responsible research for food chemists studying native Australian plant foods involves upholding the rights of Indigenous Australians, working with communities when the foods grow on their land, complying with international treaties, following laws and obtaining permits and benefit sharing agreements where required, and working ethically toward and with all stakeholders.

## Data Availability Statement

The original contributions presented in the study are included in the article/supplementary material, further inquiries can be directed to the corresponding author.

## Author Contributions

SF, HES, and YS conceptualized the paper. SF researched and wrote the paper. HES, HJS, MR, and YS supervised the project. SF, HES, HJS, MR, and YS edited the paper. All authors contributed to the article and approved the submitted version.

## Funding

This research was jointly funded by the Australian Government through the Australian Research Council Industrial Transformation Training Centre for Uniquely Australian Foods Grant number IC180100045, the Queensland Government Department of Agriculture and Fisheries and the University of Queensland. SF's Ph.D. is supported by an Australian Government Research Training Program Scholarship and the University of Queensland.

## Conflict of Interest

The authors declare that the research was conducted in the absence of any commercial or financial relationships that could be construed as a potential conflict of interest.

## Publisher's Note

All claims expressed in this article are solely those of the authors and do not necessarily represent those of their affiliated organizations, or those of the publisher, the editors and the reviewers. Any product that may be evaluated in this article, or claim that may be made by its manufacturer, is not guaranteed or endorsed by the publisher.
